# Medication adherence in the elderly population with chronic diseases: a factor analysis 

**DOI:** 10.3389/fphar.2025.1699999

**Published:** 2025-12-12

**Authors:** Doris Cardona, Valeria Santacruz-Restrepo, Alejandra Rendón-Montoya, Juliana Madrigal-Cadavid, Alejandra Segura-Cardona, Jorge Iván Estrada-Acevedo

**Affiliations:** 1 Independent Researcher in a Postdoctoral Fellowship, Medellín, Colombia; 2 Young Researcher and Innovator. HelPharma, Medellín, Colombia; 3 Pharmacoepidemiology and Risk Management Group, HelPharma, Medellín, Colombia; 4 Faculty of Psychology, Universidad CES, Medellín, Colombia

**Keywords:** medication adherence, aging, chronic disease, medication therapy management, SDG3, health and wellbeing, universal health coverage

## Abstract

**Background:**

Elderly adults suffer from one or more high-cost chronic diseases, consume more medications; therefore, adherence to prescribed treatments is essential to ensure effective management of these conditions.

**Objective:**

To identify the characteristics that most significantly affect medication adherence among older adults in Colombia.

**Methods:**

This was a quantitative, cross-sectional study with an analytical intent based on all medical records, medication orders, dispensing, and pharmacotherapeutic follow-up of patients over 65 years of age treated by a pharmaceutical manager between 2019 and 2025. Medication adherence was related to certain sociodemographic, clinical, and pharmacological characteristics. The analyses were univariate, bivariate, and multivariate, using an explanatory model to control for potentially confounding variables (with a significance level greater than 5%), crude and adjusted proportion ratios, 95% confidence intervals, and factor analysis using the principal component method.

**Results:**

A total of 42,601 records of older adults were evaluated. Medication non-adherence was 5.9%, and pharmacological factors were the most statistically associated, mainly problems of inappropriate use; suffering from various chronic diseases with various pharmacological regimens, coupled with administrative inefficiencies and inappropriate use of medications, are variables that influence non-adherence.

**Conclusion:**

From a public health perspective, medication adherence in older adults is a determinant of health outcomes and a key factor for the sustainability of health systems. Optimizing adherence requires comprehensive interventions framed within public policies that guarantee continuity of care, timely access to medications, and the strengthening of interdisciplinary teams. Recognizing adherence as a collective, and not just an individual, challenge is essential to reducing preventable complications, lowering hospitalization rates, and improving the quality of life of an increasingly aging population.

## 1 Introduction

Medication adherence is the autonomous behavior adopted by an individual regarding medication use, complying with the medical recommendations provided (Silva et al., 2022; Maniki et al., 2024). These conditions are permanent in nature and require professional guidance for their proper management (World Health Organization, 2025). Long-term adherence also includes attending medical appointments, following the recommendations of healthcare professionals, maintaining a healthy diet, making changes to adopt a healthier lifestyle, getting vaccinated, and ensuring the administration of prescribed treatments, among other actions; therefore, the patient’s relationship with healthcare professionals (Religioni et al., 2025) and pharmacists (Farhana et al., 2025) is key.

Although recent studies indicate that there is no single, universally accepted method for measuring adherence, and its estimation depends on the technique used—subjective measurements (self-report, questionnaires, scales) or objective measurements (pill counts, electronic monitoring, plasma levels, pharmacy records)—and in older adults, both categories are combined (Miyazaki et al., 2024; Rohay and Dunbar-Jacob, 2024).

Medication adherence is a process by which patients take their medication as prescribed, and it consists of the following phases: initiation, administration, and discontinuation. For this study, the first phase of the process was considered: initial or primary medication non-adherence (PMN), which occurs when a new medication is prescribed, but the patient does not receive it, or when an appropriate alternative is considered, within an acceptable timeframe after the prescription (Adams and Stolpe, 2016).

With the aging population and increased life expectancy, the development of chronic diseases is becoming more frequent, which in turn leads to greater use of multiple medications (polypharmacy) and increasingly complex treatment regimens (Rodríguez and García, 2018). This increases the risk of treatment non-adherence, with both clinical and economic consequences for elderly patients (Polat et al., 2020), their families, and the healthcare system, due to the high costs associated with treatment failure (Dusetzina et al., 2023; Poonawalla et al., 2024). Older adults experience not only multimorbidity—defined as the coexistence of two or more chronic diseases in an individual and associated with a higher risk of premature death, functional decline, poor quality of life, and increased use of medical services (Skou et al., 2022)—but also geriatric syndromes such as depression, constipation, chronic pain, polypharmacy, dysphagia, low weight, hypoalbuminemia, and functional limitations (Iwakiri et al., 2024).

Among other factors associated with adherence to pharmacological treatment in the management of chronic diseases in older adults are demographic factors such as sex, educational level, marital status, and occupation (Polat et al., 2020); as well as personal attitudes and beliefs (Lee et al., 2018) and difficulties opening bottles or swallowing. Factors that influence adherence to treatment include the excessive size of the pills, lack of understanding of the treatment, lack of knowledge about the disease, forgetfulness, memory loss, transportation difficulties, and long wait times.

## 2 Materials and methods

This project was framed within a quantitative, descriptive, observational approach with an analytical intent (not aimed at establishing causality), and a cross-sectional design. Medication adherence among patients with chronic diseases was analyzed using secondary data based on medical records, medication orders, dispensing, and pharmacotherapeutic follow-up of 42,601 people over the age of 65 who were part of a drug safety program at a pharmaceutical healthcare provider in Colombia between 2019 and 2025. Using big data and advanced analytics tools, the variables of interest from each of the databases were cross-referenced into a single database for final analysis. The data are not publicly available and can be provided with prior authorization from legal representatives.

The dependent variable, medication adherence, was operationalized as a qualitative, dichotomous variable (non-adherent/adherent), considering primary medication nonadherence (PMN), when a new medication is prescribed, but the patient does not obtain the medication, or an appropriate alternative, within an acceptable period of time after it was prescribed (within 30 days of the date it was first prescribed) (Adams and Stolpe, 2016). The independent variables were classified according to the factors under study.

- The sociodemographic factor consisted of 5 variables. These variables primarily related to individual and contextual characteristics, such as age, sex, marital status, education level, and socioeconomic status. Variables for this sociodemographic factor, with more than 30% missing data, were not included in the factor analysis. This absence could be explained by the pharmaceutical manager’s need for data primarily related to medications.
- The clinical factor refers to the health conditions of the elderly patients recorded in the data sources and consisted of 7 characteristics related to health status, diagnoses, polypharmacy, and other related aspects.
- The pharmacological factor is composed of 7 variables related to medication use, prescribing errors, inappropriate use problems, administrative and pharmacological inefficiencies.

“Polypharmacy” was defined as the regular intake of five or more medications (Dovjak, 2022); “adverse drug reactions” as a noxious and unintended response to a medicine, excluding poisoning or accidental or intentional overdoses (Bellanca et al., 2023; European Medicines Agency, 2025). “Prescribing errors” were classified as inappropriate prescriptions that may lead to medication safety issues or toxicity (including contraindicated drugs, lack of renal or hepatic dose adjustment when required, unnecessary chronic use of a medication, duplication, and drug–drug interactions). “Pharmacological inefficiencies”, on the other hand, referred to prescribing errors that may affect or compromise therapeutic effectiveness (incorrect dose or frequency leading to underdosing or overdosing, omission of a necessary prescription, or inappropriate pharmaceutical form).

Meanwhile, “inappropriate use problems” were defined as administration or use errors by patients or their caregivers (self-medication, incorrect dosage or frequency, missed doses, improper administration technique, among others). Finally, “administrative inefficiencies” referred to difficulties in the authorization or dispensing of medications due to administrative processing errors or issues related to the patient’s institutional affiliation with the General Social Security System for Health, according to *Law 100 of 1993* (Congress of the Republic, 1993), the comprehensive social security system aims to guarantee the inalienable rights of individuals.

The data were processed using univariate, bivariate, and multivariate analyses. The results are presented in scientific tables. To identify the sociodemographic, clinical, and pharmacological characteristics of elderly patients with high-cost chronic diseases, proportions were calculated as frequency measures for qualitative variables, and descriptive statistics, such as measures of central tendency (mean), position (median, mode, and quartiles), and dispersion (standard deviation), were used for quantitative variables. To establish the relationship between sociodemographic, clinical, and pharmacological characteristics and medication adherence, bivariate and multivariate analyses were used. In the bivariate analysis, the Chi-square statistical test was used. Statistical association was considered for tests with a significance level below 5%, and the analysis was complemented with epidemiological measures such as crude (cPR) and adjusted (aPR) proportion ratios (PR), along with their 95% confidence intervals (95% CI), in order to control for potentially confounding variables, mainly age and sex, which were included in all analyses. Additionally, in the adjusted models, sociodemographic variables with coverage error (missing data) greater than 30% and that did not show statistical association in the crude measures were excluded.

All variables that showed a statistical association in the bivariate analysis were included in a factor analysis using the principal components method to identify the variables with the greatest impact on nonadherence, considering correlations greater than 0.40. Oblimin rotation was applied, and the Kaiser-Meyer-Olkin (KMO) test and Bartlett’s sphericity test were used to assess model adequacy.

## 3 Results

### 3.1 Sociodemographic characteristics of elderly adults related to medication adherence

A total of 42,601 records of older adults were analyzed, revealing a medication adherence rate of 94.1% (n = 40,086). Among the older adults studied, those aged 70–79 years represented 43.5% (n = 18,519), with no statistically significant differences between non-adherent (44.6%) and adherent (43.4%) individuals. A higher tendency toward non-adherence was observed at younger ages; for instance, among those aged 65–69 years, the probability of non-adherence increased by 32% (PR = 1.32; 95% CI: 1.07–1.64) compared with individuals aged 90 years and older. Sex did not show statistically significant differences. Interestingly, these two sociodemographic variables—age and sex—did not demonstrate strong associations with adherence but acted as confounding variables in subsequent analyses. Individual and contextual characteristics related to medication adherence are presented in Table 1.

**TABLE 1:** Sociodemographic chracteristics associated with medication adherence in older adults.

Sociodemographic characteristics	Medication adherence				χ^2^	p-value	PR	95%CI	
	Non-adherent (n = 2515)		Adherent (n = 40086)						
	n	%	n	%				LL	UL
Age group									
65–69	751	29.9	11,779	29.4	7.88	0.05	1.32	1.07	1.64
70–79	1,121	44.6	17,398	43.4			1.34	1.09	1.65
80–89	541	21.5	8,793	21.9			1.28	1.03	1.59
90+	102	4.1	2,116	5.3			1.00		
Sex									
No data	0	0.0	14	0.0	0.15	0.70			
Male	930	37.0	14,665	36.6			1.02	0.94	1.11
Female	1,585	63.0	25,407	63.4			1.00		
Education level									
No data	750	29.8	12,920	32.2	17.20	0.00^a^			
None	573	22.8	9,722	24.3			1.00		
Primary	336	13.4	4,251	10.6			1.34	1.17	1.54
Secondary	692	27.5	10,612	26.5			1.12	0.99	1.24
Higher education	154	6.1	2,434	6.1			1.07	0.89	1.29
Postgraduate	10	0.4	147	0.4			1.15	0.61	2.20
Marital status									
No data	754	30.0	12,930	32.3	1.69	0.43			
Single/Separated	715	28.4	10,735	26.8			1.00		
Married/Common-law union	876	34.8	13,932	34.8			0.94	0.85	1.05
Widowed	170	6.8	2,489	6.2			1.03	0.86	1.22
Socioeconomic stratum									
No data	1,247	49.6	22,480	56.1	8.79	0.01^a^			
Low (1–2)	782	31.1	10,278	25.6			1.94	1.13	3.32
Medium (3–4)	472	18.8	6,972	17.4			1.72	1.00	2.96
High (5–6)	14	0.6	356	0.9			1.00		

^a^ Statistically significant association (p-value <0.005); PR: crude prevalence ratio; χ2: chi-square statistical test; 95CI: 95% confidence interval; LL: lower limit; UL: upper limit.

The same applies to marital status, which does not show a statistically significant association (p = 0.43), but does reveal a high proportion of older adults who are married or in a common-law union, with a higher percentage observed in both non-adherent and adherent groups. The level of education did show an association with medication adherence; secondary education was the most frequent, and one-fourth had no recorded level of instruction. However, older adults with only a primary education were more likely to be non-adherent (PR = 1.34; 95% CI: 1.17–1.54).

The socioeconomic status of the households of elderly adults show a high rate of underre-porting, with more than half of the records lacking this information (55.7% missing data). The type of housing in which the older adult resides showed a statistically significant association (p = 0.01). In socioeconomic low socioeconomic housing (strata 1 and 2), the likelihood of non-adherence in-creased by 94% (PR = 1.94; 95% CI: 1.13–3.32).

### 3.2 Clinical characteristics of elderly adults related to medication adherence

The health system characteristics found to be related to medication adherence in elderly adults with chronic diseases include the type of health insurance affiliation, the Health Promotion Entity (EPS) responsible for their care, and whether they had a complementary insurance/plan. Clinical variables associated with adherence include multimorbidity, polypharmacy, use of hospitalization and emergency services, and functional capacity Table 2.

**TABLE 2:** Clinical and health system characteristics associated with medication adherence in older adults.

Clinical and health system characteristics	Medication adherence				χ^2^	p-value	PR	95%CI	
	Non-adherent (n = 2515)		Adherent (n = 40086)						
	n	%	n	%				LL	UL
Health system affiliation									
Contributor	2,284	90.8	36,811	91.8	27.08	0.00^a^	1.00		
Beneficiary	90	3.6	1,796	4.5			0.81	0.65	1.00
Subsidized	141	5.6	1,479	3.7			1.54	1.29	1.84
Complementary insurance									
Yes	158	6.3	3,437	8.6	15.94	0.00^a^	1.00		
No	2,357	93.7	36,649	91.4			1.40	1.19	1.65
Multimorbidity (2+)									
Yes	1,368	54.4	21,290	53.1	1.56	0.21	1.05	0.97	1.14
No	1,147	45.6	18,796	46.9			1.00		
Out-of-target lab results									
Yes	929	36.9	14,053	35.1	3.67	0.05	1.09	0.99	1.18
No	1,586	63.1	26,033	64.9			1.00		
Hospitalizations in the last year									
Yes	416	16.5	6,505	16.2	0.17	0.68	1.02	0.92	1.14
No	2,099	83.5	33,581	83.8			1.00		
Emergency visits in the last year									
Yes	101	4.0	1,516	3.8	0.36	0.55	1.06	0.87	1.31
No	2,414	96.0	38,570	96.2			1.00		
Polypharmacy (≥5)									
Yes	661	26.3	11,441	28.5	5.93	0.02^a^	0.89	0.82	0.99
No	1,854	73.7	28,645	71.5			1.00		

^a^ Statistically significant association (p-value <0.005); PR: crude prevalence ratio; χ2: chi-square statistical test; 95CI: 95% confidence interval; LL: lower limit; UL: upper limit.

This analysis showed a statistically significant association (p < 0.005) between medication adherence and the type of affiliation to the social security health system, possession of a complementary insurance/plan, the EPS providing care, and whether the older adult was on polypharmacy. Percentage differences were observed between non-adherent and adherent individuals across both the contributory regime (contributors and beneficiaries) and the subsidized regime. It is within the subsidized category that the likelihood of being non-adherent increases by 53.5% (PR = 1.54; 95% CI: 1.29–1.84). Older adults without a health insurance policy or complementary plan accounted for 91.6% (n = 39,006), with a two-percentage-point higher proportion of non-adherent individuals compared to adherent ones. In this group, the likelihood of non-adherence increased by 39.9% (PR = 1.4) compared to patients who had a policy or complementary plan.

Of the older adults analyzed in these records, 53.2% (n = 22,658) were classified as having multimorbidity. Although no statistically significant differences were found in adherence levels (p = 0.21), having multiple simultaneous conditions (two or more) was associated with a 5.3% higher likelihood of non-adherence. This variable was included in the adjusted analysis, given that existing literature supports its association with medication adherence. Similarly, 28.4% (n = 12,102) of the individuals were classified as taking five or more medications (polypharmacy). However, those taking fewer than five medications were more likely to be non-adherent (PR = 1.12), with a statistically significant association.

No statistically significant association was found between medication adherence and the use of healthcare services, such as hospitalizations or emergency visits in the past year. Among the older adults analyzed, 16.2% had been hospitalized and 3.8% had visited emergency services. However, those who had received care in both settings showed a higher likelihood of being non-adherent to treatment. The low frequency of older adults with some form of limitation, disability, or functional impairment is noteworthy.

### 3.3 Pharmacological characteristics of elderly adults related to medication adherence

The pharmacological characteristics that showed a statistically significant relationship with medication adherence include the improper use of medications (prescribing errors and inappropriate use problems), as well as failure to collect prescribed treatments in a timely manner (persistence) (Table 3). Older adults who were persistent with their pharmacological treatments accounted for 85.5% (n = 36,439), with a 2.5% higher proportion among adherent individuals. However, those who were not persistent (i.e., did not collect their medications) had a 22.9% higher probability of being non-adherent (PR = 1.23; 95% CI: 1.10–1.37). Additionally, 83.2% (n = 35,462) of the medications dispensed were from the Basic Health Plan (PBS), with no statistically significant association observed with non-adherence to pharmacological treatment (p = 0.10).

**TABLE 3:** Pharmacological characteristics associated with medication adherence in older adults.

Pharmacological characteristics	Medication adherence				χ^2^	p-value	PR	95%CI	
	Non-adherent (n = 2515)		Adherent (n = 40086)						
	n	%	n	%				LL	UL
Persistence									
Yes	2,087	83.0	34,352	85.7	14.04	0.00^a^	1.00		
No	428	17.0	5,734	14.3			1.23	1.10	1.37
Medication dispensing (PBS)									
Yes	2,064	82.1	33,398	83.3	2.64	0.10	1.00		
No	451	17.9	6,688	16.7			1.09	0.98	1.21
Administrative inefficiencies									
Yes	200	8.0	1,721	4.3	71.13	0.00^a^	1.93	1.65	2.24
No	2,315	92.0	38,365	95.7			1.00		
Prescribing errors									
Yes	256	10.2	5,245	13.1	17.65	0.00^a^	0.75	0.66	0.86
No	2,259	89.8	34,841	86.9			1.00		
Inappropriate use problems									
Yes	886	35.2	2,284	5.7	2188.07	0.00^a^	9.00	8.21	9.87
No	1,629	64.8	37,802	94.3			1.00		
Pharmacological inefficiencies									
Yes	556	22.1	15,885	39.6	291.27	0.00^a^	0.43	0.39	0.48
No	1,959	77.9	24,201	60.4			1.00		
Adverse drug reaction (ADR)									
Yes	143	5.7	2,656	6.6	3.40	0.07	0.85	0.71	1.01
No	2,372	94.3	37,430	93.4			1.00		

^a^ Statistically significant association (p-value <0.005); PR: crude prevalence ratio; χ2: chi-square statistical test; 95CI: 95% confidence interval; LL: lower limit; UL: upper limit.

Administrative inefficiencies were present in 4.5% of the cases, with a higher percentage observed among non-adherent individuals (8%) compared to adherent ones (4.3%). Among those experiencing administrative inefficiencies, the likelihood of non-adherence increased significantly (PR = 1.93). Similarly, 12.9% of older adults experienced prescribing errors. However, notably, this situation was associated with a reduced likelihood of non-adherence, decreasing the risk by 24.7% (PR = 0.75; 95% CI: 0.66–0.86). The most frequent types of prescribing errors associated with non-adherence included: pharmacological duplication (144/256), chronic use of treatment (43), unnecessary medication (24), missing medication (16), and medication not indicated for condition (7). Both conditions related to prescribing errors showed a statistically significant association (p < 0.001).

Inappropriate use problems were present in 7.4% (3,170) of the cases, with notable differences between non-adherent individuals (35.2%) and adherent individuals (5.7%). Therefore, this variable receives the most attention in this study, as it represents the highest risk, though without a causal relationship, of non-adherence to the pharmacological treatment prescribed by healthcare professionals (PR = 9.00; 95% CI: 8.21–9.87). Statistically significant association was found between these two variables (p < 0.001). The most frequent types of inappropriate medication use among non-adherent individuals are primarily grouped into five categories, accounting for 94.1% (834/886) of the cases: dose omission (57.3%), treatment discontinuation (22.7%), inappropriate frequency and skipping medical appointments (6.0% each), failure to undergo medical tests (2.1%), with eight other types that have lower percentages.

Pharmacological inefficiencies was also observed in 38.6% (16,441) of cases, being more frequent among adherent individuals (39.6%) than among non-adherents (22.1%). These differences were statistically significant, and the presence of inefficiencies reduces the likelihood of non-adherence to pharmacological treatment by 56.8% (PR = 0.43). The most frequent causes of non-adherence were incorrect dosage (295/556), incorrect frequency (114), and overdosage (30).

Another pharmacological characteristic associated with non-adherence is adverse drug reactions (ADR), which were observed in 6.6% of the population (2,799 cases), without a statistically significant difference between non-adherent and adherent individuals. Among the non-adherent patients who experienced ADR, 80.4% had only one reaction.

### 3.4 Sociodemographic, clinical, and pharmacological characteristics explaining non-adherence to medications

When analyzing all sociodemographic, clinical, and pharmacological characteristics that showed a statistical association with medication adherence, particularly those with a significant influence on non-adherence (not taking prescribed medications), collinearity among independent variables was first assessed and controlled. Subsequently, a logistic regression was conducted to estimate an explanatory model that allowed for adjustment and control of potentially confounding variables in the final model. The variables included in the final multivariate model are shown in the following Table 4.

**TABLE 4:** Crude and adjusted prevalence ratios of sociodemographic, clinical, and pharmacological characteristics associated with non-adherence to medications in older adults.

Related variables	p-value	cPR	95%CI		p-value	aPR	95%CI	
			LL	UL			LL	UL
Age group								
65–69	0.05	1.32	1.07	1.64	0.19	1.27	0.99	1.63
70–79		1.34	1.09	1.65		1.22	0.96	1.56
80–89		1.28	1.03	1.59		1.14	0.89	1.47
90+		1.00				1.00	-	-
Sex								
Male	0.70	1.02	0.94	1.11	0.71	1.02	0.92	1.14
Female		1.00				1.00	-	-
Education level								
None	0.00^a^	1.00			0.01^a^	1.00	-	-
Primary		1.34	1.17	1.54		1.21	1.04	1.40
Secondary		1.12	0.99	1.24		0.96	0.85	1.08
Higher education		1.07	0.89	1.29		0.88	0.72	1.07
Postgraduate		1.15	0.61	2.20		0.98	0.50	1.93
Health system affiliation								
Contributor	0.00^a^	1.00			0.01^a^	1.00	-	-
Beneficiary		0.81	0.65	1.00		0.87	0.52	1.45
Subsidized		1.54	1.29	1.84		1.48	1.19	1.84
Complementary insurance								
Yes	0.00^a^	1.00			0.00^a^	1.00	-	-
No		1.40	1.19	1.65		1.59	1.30	1.94
Polypharmacy (5+)								
Yes	0.02^a^	0.89	0.82	0.98	0.48	0.95	0.83	1.09
No		1.00				1.00	-	-
Multimorbidity (2+)								
Yes	0.21	1.05	0.97	1.14	0.70	1.02	0.91	1.16
No		1.00				1.00	-	-
Persistence								
Yes	0.00^a^	1.00	-	-	0.00^a^	1.00	-	-
No		1.23	1.10	1.37		0.78	0.68	0.90
Administrative inefficiencies								
Yes	0.00^a^	1.93	1.65	2.24	0.01^a^	1.30	1.06	1.59
No		1.00	-	-		1.00	-	-
Prescribing errors								
Yes	0.00^a^	0.75	0.66	0.86	0.00^a^	0.66	0.56	0.77
No		1.00	-	-		1.00	-	-
Inappropriate use problems								
Yes	0.00^a^	9.00	8.21	9.87	0.00^a^	8.83	7.90	9.85
No		1.00	-	-		1.00	-	-
Pharmacological inefficiencies								
Yes	0.00^a^	0.43	0.39	0.48	0.00^a^	0.43	0.38	0.48
No		1.00	-	-		1.00	-	-

^a^ Statistically significant association (p-value <0.005); cPR: crude prevalence ratio; aPR: adjusted prevalence ratio; 95%CI: 95% confidence interval; LL: lower limit; UL: upper limit.

Some sociodemographic variables lost their statistical association, but some were adjusted for association with the remaining variables that showed statistical association in the bivariate analysis. Among them, age and sex groups, for example, only primary school education showed a strength of association of 21% (PR = 1.21).

Multimorbidity was a clinical characteristic included in the final model, where it reduced the probability of non-adherence from 5% in the crude analysis (cPR = 1.05) to 2% (aPR = 1.02), compared to older patients with multiple concurrent chronic conditions. It is worth noting that this latter variable did not show a statistical association in the bivariate analysis, but it was included in the multivariate analysis, since more than half of the patients suffer from more than two diseases, which has been theoretically linked to non-adherence to treatment. Authors such as Hernán and Robins (2020) suggest not relying solely on statistical criteria for variable inclusion in multivariate models. Likewise, polypharmacy maintained its statistical association (p = 0.00) when controlling for all variables in the final explanatory model, indicating that adults taking fewer than five medications have a 5% higher probability of non-adherence (cPR = 0.95).

Pharmacological variables contributed the most to the final explanation of non-adherence, as they showed statistical association in both the crude and adjusted prevalence ratios. In this way, six variables were included in the final model: persistence, adverse drug reaction (ADR), administrative inefficiencies, prescribing errors, inappropriate use problems, and pharmacological inefficiencies. Non-persistence or failing to collect medications on time on the scheduled dates, showed a strong association in the bivariate analysis (cPR = 1.23), but in the multivariate model, it reduced the likelihood of non-adherence by 22% (aPR = 0.78), indicating a change in direction between the two analyses.

Finally, the misuse of medications (prescribing errors, inappropriate use problems, as well as pharmacological and administrative inefficiencies) maintained a statistically significant association after controlling all associated variables simultaneously in the multivariate model. In this way, the five variables related to how older adults manage medications for high-cost chronic conditions decrease in strength of association in the adjusted analysis but maintain their original positive or negative direction. Therefore, problems resulting from the inappropriate use of treatments are the characteristic that most strongly explains non-adherence, after controlling for all variables (Adjusted Prevalence Ratio cRP = 8.83; 95% CI: 7.90–9.85).

Finally, a factor analysis was conducted using the principal components method with Varimax rotation for better model adjustment. The adequacy of the factor analysis was assessed using the Kaiser-Meyer-Olkin index (KMO = 0.504). Although it is within the acceptable lower limit, it is considered sufficient to continue with the proposed exploratory factor analysis. There is a significant correlation between the variables, according to Bartlett’s test of sphericity (p = 0.000), justifying the factor model. Furthermore, the model explained 59.1% of the total variance, close to the recommended threshold of 60% for a model with acceptable explanatory power (Hair et al., 2019). The remaining variance is explained by other characteristics.

According to the matrix rotated with Oblimin, five (5) factors were found that explain the variability of non-adherence in 59.16%, made up of 10 characteristics: clinical (polypharmacy and multimorbidity), administrative procedures (administrative inefficiencies and inappropriate use problems), access to the health system (affiliation to the system), demographic (age group and having a health plan) and pharmacological (pharmacological inefficiencies and persistence) Table 5.

**TABLE 5:** Principal components for non-adherence to medications in older adults.

Characteristics	Principal component				
	Clinical	Administrative procedures	Healthcare access	Demographics	Pharmacological
Multimorbidity (2+)	0.882				
Polypharmacy (5+)	0.851				
Administrative inefficiencies		0.610			
Sex		0.592			
Inappropriate use problems		−0.513			
Health system affiliation			0.846		
Age groups				0.753	
Complementary insurance				0.653	
Pharmacological inefficiencies					0.815
Persistence					0.429

Extraction method: principal component analysis; Rotation method: Oblimin with Kaiser normalization; KMO, 0.504; Bartlett’s test of sphericity = 0.000; Total variance explained = 59.1%.

## 4 Discussion

Non-adherence to medications for the treatment of chronic diseases among older adults in this study was 5.9%, similar to the findings reported by Aznar-Lou et al. (2017), the prevalence of initial medication non-adherence (IMNA), defined as the failure to obtain a medication the first time it is prescribed, was 17.6% of prescriptions, varying between 7.4% and 22.6% and Spadea et al. (2021) found low rates of nonadherence, ranging from 8% to 13% for prescribed drug therapies. A comparable of 2.8% was reported by Polat and collaborators (Polat et al., 2020) regarding the lack of motivation for medication adherence. However, this is much lower than the 69.9% non-adherence rate found among Ecuadorian adults with chronic diseases by Padilla-Vinueza and Morales-Solis (2020) and 31.8% the patients aged 65 years or older with multimorbidity and polypharmacy (Liu et al., 2023). Similarly, in the main chronic conditions, adherence to treatment varies from 57.6% in arterial hypertension in Perú (Silva Fhon et al., 2024) 55.9% in China (Lo et al., 2016), and 46%–100% in oral cancer (Mislang et al., 2017).

Good adherence has been estimated at 80%, but adherence is a process that involves three major phases, during which treatment abandonment can occur: initiation (from the time of prescription to the first dose), implementation (proportion of prescribed medications taken, number of days with medications taken, doses taken on time, etc.), and discontinuation (skipping doses and interrupting treatment). Therefore, the time between initiation and the last dose before discontinuation is considered persistence, and non-persistence accounts for 50% of non-adherence among patients with hypertension during the first year of treatment (Burnier and Egan, 2019).

Demographic factors such as younger age among older adults and having only secondary-level education are associated with a higher risk of poor medication adherence, primarily due to the increased incidence of chronic diseases and polypharmacy among individuals over 65 years of age (Liquori et al., 2022). A similar finding was observed in the present study; it is worth noting that these two characteristics did not show a statistically significant association in the adjusted models, but they did demonstrate specific weight and contributed to explaining the variability in non-adherence, as identified in the principal components analysis. Other characteristics that showed a statistically significant association with non-adherence, consistent with the findings of this study, included male sex, and residence in low socioeconomic strata among patients with type 2 diabetes mellitus, hypertension, and heart failure (Steve et al., 2021). Contrary to the findings reported by Polat and collaborators (2020) where men and employed professionals showed higher levels of motivation for medication adherence.

The rural area of origin was also found to have a strong association, though not statistically significant, indicating that living in rural areas is a barrier to adherence to pharmacological treatments—similar to the finding reported by Mamaghani et al. (2020) in hypertensive patients, other also found that the duration of chronic diseases is associated with therapeutic adherence (Chu et al., 2021), possibly due to patients’ learning and increased understanding of their own diseases over time.

Multimorbidity is a clinical factor that was found to be statistically associated with non-adherence (an increase of 24.9%), similar to findings in other studies (Foley et al., 2023). A higher number of prescribed medications for hypertension, has also been associated with non-adherence medication (Ruksakulpiwat et al., 2024), contrary to the findings of this study, where polypharmacy was identified as a possible factor that promotes adherence. It is noteworthy that multimorbidity and polypharmacy are the variables that contribute most to the first principal component and are the main drivers of the variability in medication non-adherence in this study.

Older adults suffer from multiple chronic conditions (multimorbidity), which leads to the simultaneous use of various pharmacological treatments (polypharmacy), a situation that exposes them to a higher risk of experiencing Medication-related problems (Pramotesiri et al., 2024). In the present study, Medication-related problems were defined as prescribing errors, inappropriate use problems, pharmacological and administrative inefficiencies. These characteristics, which belong to the pharmacological factor, showed a strong association, explained the variability, and were the most significant contributors to non-adherence among older adults with non-communicable chronic conditions. Many of these issues are preventable through healthcare interventions and pharmaceutical management, particularly via careful medication prescribing practices (Murphy et al., 2022).

Other variables that showed a statistically significant association with non-adherence were affiliation to the social security health system and having private or complementary health insurance plans. Those who are part of the subsidized healthcare regime and do not have additional health plans beyond the basic coverage were more likely to abandon treatment. It should be acknowledged that this is a situation specific to Colombia and may not be fully comparable to findings from other studies. For example, Al Bawab et al. (2021) found that the frequency of medication intake and having health insurance were factors that decreased medication adherence among Jordanian patients with chronic diseases.

This particular situation regarding barriers to accessing health services in Colombia had already been described by the Pan American Health Organization (2023) (Chapter 3). Mainly due to long waiting periods and the complexity of service approval processes, including equal treatment and opportunities in access to health promotion, prevention, diagnosis, treatment, rehabilitation, and palliative care activities. This is in accordance with Article 49 of the Colombian Political Constitution, and any deviation from this standard constitutes a violation of the right to health, especially for older adults, who are subjects of special protection as established in Article 11 of Statutory Law 1715 (Congress of the Republic, 2015), similar to target 3.8 of Sustainable Development Goal 3 (SDG 3: Good health and wellbeing “Ensure healthy lives and promote wellbeing for all at all ages”), related to “Universal health coverage: “Achieve universal health coverage, including financial risk protection, access to quality essential healthcare services and access to safe, effective, affordable and quality essential medicines and vaccines for all” (United Nations, 2015).

The inappropriate use problems and prescribing errors were pharmacological characteristics found to be statistically associated with non-adherence. These, in turn, are causes of adverse events related to medication use; therefore, they are conditions that should be studied in parallel (Stanly et al., 2025) since they lead to adverse reactions, medication-related problems, and pharmacological inefficiencies. As observed in the present study; in addition to polypharmacy, which also causes undesirable events for the patient (Dovjak, 2022).

Finally, the findings of the present study are consistent with other studies. These have also shown that variables such as lower socioeconomic status (a sociodemographic factor) (Cummings et al., 2016) are associated with lower therapeutic adherence. Multimorbidity and polypharmacy (clinical factors) (González-Bueno et al., 2021; Selvakumar et al., 2023) as well as administrative inefficiencies and shortcomings in the health system (factors related to the provision of healthcare services) (Wang, Cheng and Li, 2024; Horvat, Eržen and Vrbnjak, 2024), also play a significant role. Together, these are the main characteristics explaining non-adherence to chronic disease treatments among older adults.

Poor adherence to pharmacological treatments in older adults represents a significant challenge for both clinical practice and public health, due to its considerable medical and economic repercussions. It is estimated that between one-fifth and more than half of older adults do not adequately follow prescribed therapeutic regimens, which undermines treatment efficacy and hinders the management of chronic diseases (Fadil et al., 2023). In geriatric patients with multiple comorbidities, lack of adherence becomes a key factor accelerating the deterioration of their health status, increasing the likelihood of decompensation and adverse clinical events. These difficulties are reflected in increased use of emergency services, frequent hospital readmissions, greater pressure on healthcare resources, and increased mortality (El-Sayed et al., 2023).

Population aging is closely linked to high rates of multimorbidity and polypharmacy, factors that substantially complicate the consistent use of medication. From an economic perspective, poor adherence places a considerable financial burden on healthcare systems, as well as on families and caregivers. Evidence shows that improving adherence could significantly reduce the costs associated with managing preventable complications (Lee et al., 2018). In many cases, family members must cope with the consequences of inadequate management of chronic diseases in older adults, assuming greater caregiving responsibilities due to adverse events such as strokes, myocardial infarctions, or progressive cardiac dysfunction events that could often be mitigated through proper treatment adherence.

In general, medication adherence in older adults with chronic diseases is a multifactorial process with profound implications for both individual and population health. Non-adherence to treatment regimens perpetuates a recurring cycle of poor disease control, the development of complications, and increased use of healthcare services (Hanna et al., 2020).

Limitations: The authors acknowledge the strength of having a large sample size, but some limitations arise: a) the cross-sectional design of the study did not allow adherence to be measured at other time points; only initial or primary non-adherence was analyzed; b) database limitations: data are secondary, institutional, and not population-representative; c) some sociodemographic variables contained missing data that could not be imputed and were therefore excluded from the bivariate and multivariate analyses. In particular, variables with a coverage error greater than 30% and no statistical association with the variable of interest were omitted. This absence may be explained by the fact that the data source was a pharmaceutical management entity responsible for medication administration, which focuses on identifying modifiable characteristics at an early stage, while non-modifiable ones are often disregarded in preventive pharmaceutical management actions; d) the fact that some variables used in this study did not replicate the patterns reported in previous studies may be attributed to significant differences in sociodemographic and clinical characteristics. These differences stem from the population being older, multimorbid, taking multiple medications, and specifically referred to this pharmacy manager; this may influence both the prevalence of adherence and the direction of specific associations, since pharmacy management involves home visits and deliveries to patients who have barriers or limitations to medication use. Furthermore, differences in the measurement of medication adherence, controlled for in the multivariate analysis, may also exist, potentially leading to patterns that do not fully align with previous findings; e) this study performed a multivariate analysis with adjustment methods for all confounding factors simultaneously; this mutual adjustment could lead to overfitting or inadequate effect estimates (Gao et al., 2025).

## 5 Conclusion

The accumulated evidence indicates that medication adherence in older adults with certain chronic diseases is a multifactorial phenomenon requiring complex, sustained, and individualized interventions. These include ensuring access to medications, recognizing cognitive, emotional, functional, social, and structural barriers, and implementing personalized strategies such as therapeutic education and simplifying treatment regimens.

Sustainability emerges as a critical dimension given that adherence must be maintained long-term throughout the lifespan of the chronic disease, health systems must be strengthened through policies that guarantee continuity of care, access to medications, patient-centered service delivery, and effective interdisciplinary collaboration. A patient-centered perspective on adherence is insufficient; it must be understood as a public health challenge requiring coordinated action among professionals, institutions, and policymakers.

Ultimately, improving adherence reduces preventable medical complications, decreases hospitalization rates, and optimizes healthcare spending. In the context of a rapidly aging global population, there is an urgent need to promote research, innovation, and the implementation of evidence-based interventions that encourage stable therapeutic behaviors, ensure adequate support for older people, and improve long-term health outcomes and quality of life.

## Data Availability

This research presents results from aggregated data available in the institutional databases of a pharmaceutical manager. Therefore, the data are not publicly accessible and may be made available upon authorization from the legal representatives. Requests to access these datasets should be directed to Helpharma S.A, jorge.estrada@zentria.com.co.

## Abbreviations

ADR, Adverse Drug Reactions; SDG, Sustainable Development Goal.
